# Statistical Association Criteria in Forensic Psychiatry–A criminological evaluation of casuistry

**Published:** 2011-02-25

**Authors:** G Costea, V Gheorghiu, O Buda, I Popescu, MS Trandafir

**Affiliations:** * ‘Mina Minovici’ National Institute of Legal Medicine, BucharestRomania; ** ‘Alexandru Obregia’ Psychiatric Hospital, BucharestRomania

**Keywords:** Antisocial behavior potential, Therapy and Risk management, Forensic assessment

## Abstract

**Purpose**. Identification of potential shared primary psychoprophylaxis and crime prevention is measured by analyzing the rate of commitments for patients–subjects to forensic examination.

**Material and method**. The statistic trial is a retrospective, document–based study. The statistical lot consists of 770 initial examination reports performed and completed during the whole year 2007, primarily analyzed in order to summarize the data within the National Institute of Forensic Medicine, Bucharest, Romania (INML), with one of the group variables being ‘particularities of the psychiatric patient history’, containing the items ‘forensic onset’, ‘commitments within the last year prior to the examination’ and ‘absence of commitments within the last year prior to the examination’. The method used was the Kendall bivariate correlation. For this study, the authors separately analyze only the two items regarding commitments by other correlation alternatives and by modern, elaborate statistical analyses, i.e. recording of the standard case study variables, Kendall bivariate correlation, cross tabulation, factor analysis and hierarchical cluster analysis.

**Results**. The results are varied, from theoretically presumed clinical nosography (such as schizophrenia or manic depression), to non–presumed (conduct disorders) or unexpected behavioral acts, and therefore difficult to interpret.

**Conclusions**. One took into consideration the features of the batch as well as the results of the previous standard correlation of the whole statistical lot. The authors emphasize the role of medical security measures that are actually applied in the therapeutic management in general and in risk and second offence management in particular, as well as the role of forensic psychiatric examinations in the detection of certain aspects related to the monitoring of mental patients.

The forensic issues related to crime and psychiatric pathology generate controversies but also lead to discussions about the shortage of inter–departmental cooperation as well as difficulties to initiate and support prevention and psychoprophylaxis programs. Similarly, both detection and monitoring/follow–up of mental patients are difficult to evaluate, because one cannot ignore the will of the patient who usually refuses any psychiatric help and assistance. Thus, it becomes extremely difficult to address the antisocial potential of mental patients and of patients without mental disorders, implying considerable logistic and conceptual efforts [1,2]. The legal provisions regarding compulsory treatment continue to be the only means by which we can ensure proper therapeutic management in a limited group of patients with antisocial potential [3,4].

## Objectives

This study proposes to emphasize the role that forensic medicine has, through forensic psychiatric examinations, in the detection of phenomena and identification of risk factors of criminal behavior, especially the violent one, and to highlight the need for an actual enforcement of legal provisions regarding compulsory treatment [5,6]. 

## Material| Method

A statistical case analysis was performed within ‘Mina Minovici’ INML, focusing on primary forensic psychiatric examination, in order to detect the medical and social phenomena of legal cases. A group variable database was created, with each of the variables consisting of several items [7]. The statistical frequency recording contained the primary forensic psychiatric examination reports, which have been completed for a full year, between 1.01.2007 and 31.12.2007.

- The above–mentioned database was used for this work, excluding the cases where the personal record data was not complete and the cases have been concluded as neurotic pathology.
- The analyzed statistical lot contains a total of 749 cases.
- This is a passive, retrospective descriptive analysis, not involving the examined person and using the data recorded in the forensic medical reports.
- The correlations between variables in the INML database were analyzed by using the Kendall method.
- Since the results have shown no correlations with the group variable ‘particularities–psychiatric patient history’, which also contains the item ‘commitments within the last year’, meaning a psychiatric commitment within the calendar year prior to the examination, the variables have been re–coded in accordance with the medical history pattern and with the specificity of a forensic psychiatric examination. Nosological classifications have been regrouped into three models for similar reasons. Another correlation was performed by using the Kendall method, which found that the new variable regarding psychiatric commitments within the year prior to the examination correlates with the following new group variables, i.e. the variable ‘minors’ (examined persons aged 14 to 17), the variable ‘adults’ (including the age group theoretically representing the active age, 18 to 64), the variable ‘65 and above’, the variable ‘mental retardation’ and the variable ‘cognitive loss’, a mixed group defining the cognitive deficit, irrespective of gravity.
- The new variable ‘commitments’ refer to patient psychiatric commitments within the year prior to the examination:
  1. As a common rule, the first forensic psychiatric examination is performed immediately after the offence has been perpetrated or after the legal proceedings have been initiated;
  2. The existence of psychiatric commitments within the last year signifies a less favorable evolution or the need for a psychiatric reassessment;
  3. The absence of commitments within the previous year implies a favourable evolution or indifference to the treatment;
  4. The perpetration of an offence settled by the law by psychotic patients or patients with cognitive deficits and with behavior or personality disorders is usually related to their antisocial potential.
- The percentages of variables with positive correlations have been calculated. Cross tabulation and calculation of the estimated risk (odd ratio) as well as hierarchical clustering have been performed. The SPSS v.10 program for Microsoft Windows has been used for the calculation.

Descriptive statistics are used to describe the main features of a collection of data in quantitative terms. Descriptive statistics are distinguished from inferential statistics (or inductive statistics), in that descriptive statistics aim to quantitatively summarize a data set, rather than being used to support inferential statements about the population that the data are thought to represent [7,8]. Even when a data analysis draws its main conclusions using inductive statistical analysis, descriptive statistics are generally presented along with more formal analyses, to give the audience an overall sense of the data being analyzed. The statistical methods, which deal with censored data, can be divided into a 2 x 2 grid: parametric vs. nonparametric, and univariate vs. multivariate. There are a number of bivariate methods, including two correlation tests and three linear regression analyses. Cox hazard model (correlation test), EM algorithm, and Buckley–James method (linear regressions) can treat several independent variables if the dependent variable contains only one kind of censoring (i.e., upper or lower limits). Generalized Kendall's tau (correlation test) and Schmitt's binned linear regression can treat mixed censoring (including censoring in the independent variable), but can only have one independent variable. 

**Figure 1:**
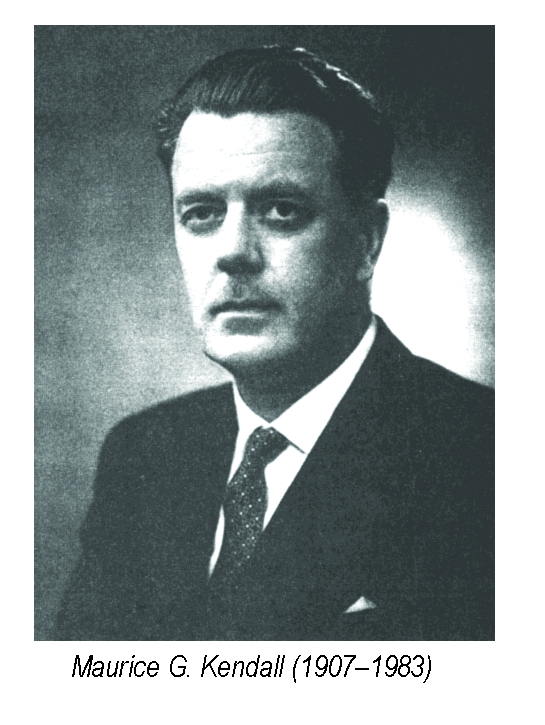
Maurice G. Kendall (1907–1983)

We propose an evaluation method based on Kendall's ।, a metric of rank correlation. The method is inexpensive, and representation independent [9,10,11]. We will show that Kendall's । reliably correlates with human behavioral ratings and reading times. The Kendall tau rank correlation coefficient (or simply the Kendall tau coefficient, Kendall's । or tau test) is a non–parametric statistic used to measure the degree of correspondence between two rankings and to assess the significance of this correspondence. In other words, it measures the strength of association of the cross tabulations [12,13,14]. It was developed by Maurice Kendall in 1938. 

## Results and Discussions

### Preliminary Statistical Data

Table 1 contains indicative preliminary basic statistical data. The apparent redundancy of certain variables is due to successive recoding, which is necessary for each type of analysis; only the most suggestive of all aspects of data dissemination have been included. Standard deviations were found to be sub–unitary. The analysis of the database created at ‘Mina Minovici’ INML indicates significant reliability both for the diagnosis and for the accurate recording of actual data;

The criminal history was found to be absent in 81.4%, the percentage cannot be attributed only to the 30.8% increase in the criminal activity among minors;

This study stems from the following picture: at ‘Mina Minovici’ INML, within the primary forensic psychiatric examination boards, roughly 800 reports are completed each year, as requested by the competent institutions, for patients usually from Bucharest; the male sex is prevalent in a 3/1 ratio, as for criminal cases, thefts followed by murder, in aggregate, acts indicative of aggressive/violent behavior, account for more than half of them; the most involved age groups are 25–44 and 14–17, which was also noticed in previous studies [9,10]; the absence of criminal history, criminal history without violence and criminal history of the same type are prevalent. The presented characteristics bring about new elements of primary prevention (81.4% without criminal history) and risk management particularities (only 28 cases with violent criminal history out of 290 violent acts committed). 

**Table 1:** Table 1

Preliminary statistical data					
	N / valid	Frequency	Percent	Mean	Std. deviation
Male	749	561	74,9	–	–
Female	749	188	25,1	–	–
Adults	749	388	51,8	1,48	0,50
Juvenile	749	231	30,8	1,69	0,46
65 years and above	749	130	130	1,83	0,38
25–44 years	749	158	21,1	1,79	0,41
18–24 years	749	117	15,6	1,84	0,36
45–64 years	749	113	15,1	1,85	0,36
Criminal record					
Penal Law Casuistry	749	507	67,8		
Theft, robbery	749	177	23,6	1,76	0,43
Homicide	749	121	16,2	1,84	0,37
Other violent crimes	749	100	13,4	1,87	0,34
Man slaughter attempts	749	69	92		
Other crimes	749	40	5,3	1,76	0,43
No offence record	749	610	81,4		
Non–violent crime record	749	111	14,8		
Violent crime record	749	28	3,7		
A record with different crimes	749	81	10,8		
Identical pattern of crime	749	57	7,6		
Psychiatric background					
Unknown psychiatric record	749	423	56,5		
Known psychiatric record	749	326	43,5		
Significant toxic inferences	749	53	7,1		
Non–psychotic behavior (conduct) disorders	749	172	23,0	1,88	0,33
Personality disorders	749	136	18,2	1,82	0,31
Dementia	749	105	14,0	1,86	0,35
Psychosis	749	103	13,8		
No mental disorders	749	91	12,1	1,88	0,33
Organic personality disorder	749	81	10,8	1,89	0,31
Mental Retardation	749	61	8,1		
Schizophrenia	749	72	9,6		
Socialized Conduct Disorder	749	164	21,9		
Unsocialized Conduct Disorder	749	8	1,1		
With previous year psychiatric commitment /confinement	326	101	13,5		

From a psychiatric perspective, most of the noticed data is theoretically presumed also by reference to the most involved age groups and gender. For future in–depth studies, one observation, which has been reiterated within the INML, is to be noted, concerning the sub–diagnosis of alcohol intake–related pathology; we must specify however that only certain data may be recorded in the forensic psychiatric examination. Which should also be noted is the fact that for a psychiatric pathology (Table 1 and Table 2 diagnosis axes) found in 658 patients, only 326 have shown psychiatric history. Of the latter, only approximately one third (101) were committed the year prior to the examination. The observations presented have supported this study, even more, so, the forensic psychiatric examination (and forensic medicine, in general) helps detect various phenomena, which may be extrapolated at a social level, and, more specifically, from the point of view of their dynamics [11]. 

### Correlation Analysis

A correlation analysis was performed on the status of last / previous year psychiatric commitments of the examined patients using the Kendall bivariate method, which found that they correlate with the following variables (Table 2 and Table 3): ‘minors’ (sig.–2 tailed = 0.006, correlation ratio 0.153, correlation type–positive, significance level 0.01), ‘adults’ (sig.–2 tailed = 0.000, correlation ratio–0.284, correlation type–negative, significance level 0.01), ‘age group 65 and above’ (sig.–2 tailed = 0.000, correlation ratio 0.226, correlation type– positive, significance level 0.01), ‘mental retardation’ (sig.–2 tailed = 0.017, correlation ratio–0.134, correlation type–negative, significance level 0.05) and ‘cognitive deficiency’ accounting for patients with mental retardation, cognitive decline irrespective of intensity, as opposed to patients with normal cognition, the variable denoting the cognitive verification of mental capacity (sig.–2 tailed = 0.038, correlation ratio 0.116, correlation type–positive, significance level 0.05).

This study only analyzed the relationships with the first two variables (‘minors’and ‘adults’) for the following reasons: they represent the patients who are mostly involved in criminal cases, and, implicitly, they are the subjects of the legal provisions regarding compulsory treatments; as a rule, cognition is within normal limits [12,13].

The ‘target’ variables of the study correlate in turn with other variables. The positive correlations of these two variables are given in Table 2 and Table 3.  

**Table 2:** Minors (Juveniles) Variable–positive correlations

Correlated Variable	N	sig.(2–tailed)	Correlation coefficient Kendall's ।b	level
Sex gender	749	0,000	0,173	0,01
Criminal record	749	0,001	0,120	0,01
Psychiatric record	749	0,001	0,505	0,01
Non–significant toxic inferences	749	0,004	0,105	0,01
Case type	749	0,000	0,448	0,01

**Table 3:** Adults Variable–positive correlations

Correlated Variable	N	sig.(2–tailed)	Correlation coefficient Kendall's ।b	level
Homicide	749	0,000	0,264	0,01
Personality disorders	670	0,000	0,370	0,01
Psychosis	670	0,000	0,263	0,01
Mental impairment	658	0,000	0,253	0,01
Sex gender	749	0,015	0,089	0,05

### ‘Minors/Juvenile’ variable

Cross tabulations and a risk estimation using the Odd Ratio calculation (Table 4) have been performed. The odds ratio is a measure of effect size, describing the strength of association or non–independence between two binary data values. It is used as a descriptive statistic, and plays an important role in logistic regression. Unlike other measures of association for paired binary data such as the relative risk, the odds ratio treats the two variables in the same way, being compared symmetrically, and can be estimated by using some types of non–random samples. Apart from target variables, ‘theft–conduct disorders’, ‘other violent acts–conduct disorders’ have also been applied to non–correlated cohorts for theoretical reasons. These variables have been chosen because minors mostly commit thefts and robberies. 

Studying the ‘theft–conduct disorder’ relationship, for N = 749 n–conduct disorders = 172, thefts were committed by 6 patients diagnosed to have non–socialized conduct disorder and by 98 patients diagnosed to have socialized conduct disorder (98 thefts by patients with conduct disorders). With regard to the reasons for forensic psychiatric examination (for N = 749–year 2007–95% confidence interval), the estimated theft risk appears to be sub-unitary, considerably higher for the socialized conduct disorder (0.912).

Studying the ‘other violent acts–conduct disorder’ relationship, for N = 749 and n valid cases of socialized conduct disorders with acts committed violently (except for murder and attempted murder) = 43, the estimated risk is above unit, 1.114. 

A cross tabulation has been attempted for the ‘commitments–conduct disorders’ relationship, but, although in theory statistical and mathematical calculations can also be made for a batch of 231 cases with only 5 cases for the analyzed item, we considered that the results cannot be very rigorous. However, we noted the following data involving discussions regarding primary prevention and psychoprophylaxis: out of 231 minor patients, 172 were diagnosed as having conduct disorder. 

Of these 231 minors, 59 already had a criminal history, and only 14 were recorded as having a psychiatric history. 

Of these 14, only 10 had been committed in a children's psychiatric hospital, 5 of whom the year prior to the examination (2 for non–socialized conduct disorder). 

The absence of referral to the psychiatric specialist network of minors with non–socialized conduct disorder indirectly implies great malfunctions in the patient's social support, the disease being perceptible and not related to the environment or the circle of family and friends. The data reflects a lack of ability in the preventive management.

Table 4 shows that the estimated risk is virtually different in minors than in other age categories in terms of the criminal history of violent acts, i.e. this risk is present only in the other age categories, being sub–unitary for minors. 

The explanation would seem logical, as minors did not have the time to commit acts that are more serious. However, if we take into account the previous observations concerning the criminal history that already exists in almost a fifth of the minors, and, given a super–unitary estimated risk of violent behavior in the same age group, we can judge that it is just a matter of time until violent acts committed by minors emerge. 

Thus, it is necessary that risk and second offence management should be considered separately for adults and teenagers, with such criteria being revised for the latter.

### ‘Adults’ Variable

Table 5 shows that the age group 18–64 is at risk of examination for any offence as well as in civil cases; both genders and virtually the entire non–neurotic pathology on both diagnosis axes are involved. This issue shows the need for other discussions regarding the risk and second–offence management evaluation criteria.

For the ‘adults’ –‘commitments’ relationship no further calculations have been made, the correlation being negative (0.01 significance level); out of N = 326 valid cases for adults with commitments, 168 patients had not been committed within the last year, while 46 had. 

A benefit of certain commitments is obvious for vulnerable adults by psychiatric pathology toward behavior punishable by the law. This is actually, strongly related to outpatient monitoring, which seems to be deficient. 

We also note data that has not been statistically analyzed due to a lack of significant correlation. Of the 72 schizophrenia patients, 68 were adults; 20 were involved in criminal cases; 54 patients had not been committed within the last year. 

According to the INML database, most of the schizophrenic patients had been examined in order to be placed under a ban by the medical confinement.

**Table 4:** Minors (Juveniles)/ Crosstabulation / Risk Estimate

Minors*male = 199	Minors*female = 32		
Other age groups*male = 362	Other age groups*female = 156		
	Value	95% Confidence Interval	
		Lower	Upper
Odds Ratio for male (male / female)	2,680	1,765	4,070
**For cohort minors = minors**	**2,084**	**1,491**	**2,913**
For cohort minor = other age groups	0,778	0,711	0,850
N of Valid Cases	749		
Minors*no criminal record = 172	Minors*with criminal record = 59		
other age groups*no criminal record = 438	other age groups*with criminal record = 80		
Odds Ratio for violent criminal records / non–violent criminal record	,378	,149	,962
**for cohort minors = minors**	**,534**	**,273**	**1,044**
for cohort minor = other age groups	1,411	1,071	1,860
N of valid cases	139		
other age groups*Unknown psychiatric record = 206	other age groups*Known psychiatric record = 312		
Odds Ratio for Unknown psychiatric record / Known psychiatric record	23,476	13,298	41,444
**for cohort minors = minors**	**11,946**	**7,096**	**20,109**
for cohort minors = other age groups	,509	,460	,563
N of valid cases	749		
Minors*Non–significant toxic inferences = 224	Minors*with Significant toxic inferences = 7		
other age groups *Non–significant toxic inferences = 472	other age groups*with Significant toxic inferences = 46		
Odds Ratio for Non–significant toxic inferences / Significant toxic inferences	3,119	1,386	7,017
**for cohort minor = minor**	**2,437**	**1,212**	**4,900**
for cohort minor = other age groups	,781	,695	,878
N of valid cases	749		
Minors*criminal case = 229	Minors*civil case = 2		
other age groups*criminal case = 279	other age groups*civil case = 239		
Odds Ratio for judicial case (penal / civil)	98,084	24,124	398,795
**for cohort minors = minors**	**54,320**	**13,618**	**216,668**
for cohort minors = other age groups	,554	,511	,600
Minors*psychiatric commitment in the previous year = 9	Minors*no psychiatric commitment, previous year = 5		
other age groups*psychiatric commitment, previous year = 92	other age groups*no psychiatric commitment, previous year = 220		
Odds Ratio for psychiatric commitment previous year / no commitment in the previous year	4,304	1,404	13,192
**for cohort minors = minors**	**4,010**	**1,379**	**11,664**
for cohort minors = other age groups	,932	,874	,993
N of valid cases	326		

**Table 5:** Adults / Crosstabulation / Risk estimate

Adults*male = 305	Adults*female = 83		
other age groups*male = 256	other age groups*female = 105		
	Value	95% Confidence Interval	
		Lower	Upper
Odds Ratio for male (male / female)	1,507	1,081	2,101
**for cohort adults = adults**	**1,231**	**1,031**	**1,471**
for cohort adults = other age groups	,817	,699	,955
N of Valid Cases	749		
Adults*homicide = 99	Adults*other crimes = 289		
other age groups*homicide = 22	other age groups*other crimes = 339		
odds ratio for omor (homicide / other crimes)	5,279	3,241	8,598
**for cohort adults = adults**	**1,778**	**1,578**	**2,003**
for cohort adults = other age groups	,337	,229	,495
N of valid cases	749		
Adults*personality disorders = 123	Adults*other = 265		
other age groups*personality disorders = 13	other age groups*other = 348		
Odds Ratio for personality disorders (Personality disorders / other)	12,425	6,863	22,493
**for cohort Adults = Adults**	**2,092**	**1,882**	**2,326**
for cohort adults = other age groups	,168	,100	,284
N of valid cases	749		
N of valid cases	749		
Adults*psychosis = 92	Adults*other = 296		
other age groups*psychosis = 11	other age groups*other = 350		
Odds Ratio for psychosis / other behavior disorders	9,889	5,193	18,833
**for cohort Adults = Adults**	**1,949**	**1,751**	**2,170**
for cohort Adults = other age groups	,197	,112	,346
N of valid cases	749		
Adults*mental retardation = 57	Adults*other = 298		
other age groups*mental retardation = 4	other age groups*other = 299		
Odds Ratio for mental retardation / other	14,298	5,123	39,905
**for cohort Adults = Adults**	**1,872**	**1,687**	**2,078**
for cohort Adults = other age groups	,131	,051	,339
N of valid cases	749		

### Hierarchical cluster analysis

Hierarchical cluster analysis was also performed. The theoretically related variables with primary and tertiary psychoprophylactic management illustrated indirectly by admitting the preceding calendar year of the psychiatric medical–legal expertise have defined a number of variables strongly correlated positively  (bivariate Kendall correlation method), at a level of significance of 0,01. The same variable is risk factor (on estimate odd ratio). The results obtained allowed us to consider risk factors in triggering the need for psychiatric medical–legal expertise and legal risk management indicators. The factors used to hierarchically cluster analysis [12,13,14]. Variables strongly correlated with ‘admissions’ were entered in this analysis and were not detailed in this study (age group 65 years and over, global cognitive deficits group). Cluster analysis data can be interpreted, taking into account the frequency as it follows:

1. According to the manner of association (the cluster schedule of cases on one variables) to obtain a picture of the person’s risk of reaching the medical - legal situation with the following hierarchy of psychiatric probabilities (presented in a descending order):
  - minors;
  - known criminal history (whether or not violent, because the frequency is higher for non–violent history) and personality disorder;
  - absence of psychotic disorders and hospitalizations in the last year, or the year an offence under the criminal law has been commited or an involvement in a civil case has been noted (expertises of that time are available);
  - age groups 65 years and older, and ‘adult’;
  - criminal cases of murder and mental retardation;
  - cognitive deficits, lack of criminal history and psychiatric history (usually show civil cases);
2. After gathering the variables, we obtained a slightly different hierarchy:
  1. Group 1: with criminal history (according to the frequency–no criminal history), psychiatric history (according to the frequency–no psychiatric history);
  2. Group 2: homicides, juveniles, with criminal history without violence, no admissions in the previous year–the group which reflects the actual facts of involvement in criminal cases of minors and persons without a history of the violent behavior and difficulties in primary psychoprophylaxis management.
  3. Group 3: male gender, type of the event and age group ‘65 year and over’. Data from this group are, as in criminal cases, linked to male and the age of ‘65 years and over’ in most of the civil cases.

## Conclusions

The presented study was performed on 749 primary forensic psychiatric examination reports registered and completed in 2007, at ‘Mina Minovici’ INML, Bucharest, with the analyzed variables being included in the database. The variable ‘commitments’, which represents the schedule of commitments of the examined patients, has been analyzed from the standpoint of commitments within the last year prior to the examination. This variable correlates at a 0.01 significance level (Kendall method) with the age groups ‘minors’, ‘adults’ (aged 18 to 64) and ‘age 65 and above’, with the variable ‘mental retardation’ and the variable ‘cognitive deficits’, which refers to the cognitive deficit, irrespective of gravity. Each of these variables correlates positively or negatively at 0.01 and 0.05 significance levels (Kendall method); this study is focused on the variables positively correlated with the ‘minors’ and ‘adults’ age groups (considered as ‘target’ for the law enforcement of articles 104, 105, and 113 of the Romanian Penal Code–C.P.). According to the hierarchical cluster analysis, the following combinations would indicate an emphasis on ‘adults’ risk age group:

- Cluster I = male, no criminal background, known psychiatric history, cognitive deficiency (regardless of severity or etiology);
- Cluster II = crime and mental retardation;
- Cluster III = male, no admissions in the previous year expertise and personality disorder;
  - Specificity request for medical –legal psychiatric expertise provided legally;
  - The assessment of minors is showing some specific dynamic social context and a possible involvement of undetected biological vulnerabilities;
  - A primary psychoprofilaxis deficit, mainly due to shortage of specialist medical staff

Correlations at a high significance level and the estimated risks referring to the relationship with any commitment the year prior to the examination prove an inability to structure the tertiary psychoprophylaxis programs in association with factors independent of the capacity of organization, such as the intrinsic progression of psychotic diseases, the impossibility of adequate monitoring determined by the patient’s refusal to receive treatment, the ethical impossibility of enforcing treatment upon these patients, medication access difficulties, etc. [14,15,16]. Under these conditions and in accordance with the patient's right to accept or refuse outpatient treatment, the legal regulations regarding compulsory treatments for persons with antisocial potential and who have committed offences punishable under the provisions of the applicable (Romanian) Penal Code–articles 104, 105 C.P., remain key elements of criminal prevention and tertiary psychoprophylaxis [17–21]. Flexible programs appear to be necessary, based on actual medical education data, which should also focus on the benefit of a yearly evaluation of disease progression following commitment of mental patients, especially of psychotic patients [22,23,26], irrespective of the nosological classification and the progress stage [25,26]. Under these conditions and in accordance with the patient's right to accept or refuse outpatient treatment, the legal regulations regarding compulsory treatments for persons with antisocial potential and who have committed offences punishable under the provisions of the applicable (Romanian) Criminal–Penal Code (C.P. art. 113, 114) remain a key elements of second offence prevention and tertiary psychoprophylaxis. In order to achieve this objective, it is recommended to use the legal means of treatment enforcement by:

- Reevaluating the risk/antisocial potential assessment criteria for persons who had behaviors punishable by the law, based on the observation of the dynamics of involved phenomena;
- The current implementation of the stipulations of Romanian Penal Code (C.P. art. 113, 114), which remain the only Law Enforcement instruments in conjunction with the forensic psychiatric specialist network.

In Romania, the actual forensic psychiatric activity is organized by the medical–legal network and the expert examinations are performed by a commission of two specialists, a psychiatrist and a coroner –being chairman of the commission [2,6,24]. The cooperation between the two networks, allows the identification of the dynamic antisocial phenomena of the persons with mental disorders on Axes I and II–DSM Diagnosis, providing an efficient understanding in the psychoprofilaxis and preventive programs.

## References

[1] Anckarsater H, Radovic S, Svennerlind C (2009). Mental disorder is a cause of crime: the cornerstone of forensic psychiatry. Int J Law Psychiatry.

[2] Tataru N, Marinov  P, Douzenis A (2010). Forensic psychiatry in some Balkan countries. Curr Opin Psychiatry.

[3] Nioche A, Pham TH, Ducro C (2010). Psychopathy and associated personality disorders: searching for a particular effect of the borderline personality disorder?. Encephale.

[4] Ogloff JR (2006). Psychopathy/antisocial personality disorder conundrum. Aust NZ J Psychiatry.

[5] Stolpmann G (2010). Forensic assessment. Are biological facts useful?. Monatsschr. fur Kriminologie und Strafrechtsreform.

[6] Scripcaru G (2002). Forensic Psychiatry.

[7] GɆb R (1996). An elementary model for statistical lot inspection and its application to sampling by variables. Metrika.

[8] Siegel S (1988). Non Parametric Statistics for the Behavioral Sciences.

[9] Lorch RF (1990). Regression analyses of repeated measures data in cognitive research. Journal of Experimental Psychology: Learning Memory, and Cognition.

[10] Kerridge D (1975). The interpretation of rank correlations. Applied Statistics.

[11] Abdi H (2007). Kendall rank correlation. Encyclopedia of Measurement and Statistics.

[12] Kendall  MG (1938). A new measure of rank correlation. Biometrika.

[13] Kendall MG (1990). Rank Correlation Methods.

[14] Lapata M (2006). Automatic Evaluation of Information Ordering: Kendall's Tau. Computational Linguistics archive.

[15] Costea G (2000). Devianta manifestata prin violenta, aspecte teoretice comparative–psihiatrice, medico–legale si juridice. Conduite autolitice–Deviante de personalitate–Recuperare in neurologia psihiatrica.

[16] Costea G (2001). Asistenţa medicală psihiatrico–medico–legală: particularităţi teoretice si practice. Coordonatele actuale ale reintegrarii bolnavului psihic.

[17] Mosescu M (2007). Implementing confinement measures from criminal code (art. 113 and 114)–practical issues. Rom J Leg Med..

[18] Arndt S (1999). Correlating and predicting psychiatric symptom ratings: Spearman's r versus Kendall's tau correlation. Journal of Psychiatric Research.

[19] Prelipceanu D (1999). Adaptare şi devianţă: o perspectivă istorică asupra psihopatiei. Revista Medicală Orădeană.

[20] Gheorghiu V (1999). Aspecte medico–legale si juridice privind efectele tardive posttraumatice. Rom J Leg Med..

[21] Costea G (2008). Medical and forensic implications in dementia. Rom J Leg Med.

[22] Coid J (2001). Medium secure forensic psychiatry services–Comparison of seven English health regions. British Journal of Psychiatry.

[23] Avramenko A (2009). Cost of care of patients with personality  disorders in forensic psychiatric hospitals in the Netherlands. Crim Behav Ment Health.

[24] Leue A (2008). Reinforcement sensitivity of sex offenders and non–offenders: an experimental and psychometric study of reinforcement sensitivity theory. Br J Psychol.

[25] Enache A (2008). Work ability evaluation in civil cases–Particular aspects. Rom J Leg Med..

[26] Timmerman IGH (2005). An integrated cognitive–behavioural approach to the aetiology and treatment of violence. Clinical Psychology and Psychotherapy.

